# 4‐Methylesculetin Ameliorates Hepatic Insulin Resistance in HepG2 Cells Through AMPK/FOXO1, PI3K/AKT/GSK3*β* Pathways and SIRT1/NOX4 Axis

**DOI:** 10.1155/jdr/8023146

**Published:** 2026-04-20

**Authors:** Xiaohua Su, Yuhang Du, Yang Yang, Yige Zhao, Hongbin Zhao, Jiamei Xie, Ziyi Shan, Menglu Wang, Zhiyun Huang, Wanxin Fu, Anfeng Wan, Yongcheng An, Baosheng Zhao

**Affiliations:** ^1^ Guangzhou Baiyunshan Xingqun Pharmaceutical Co., Ltd., Guangzhou, China; ^2^ Department of Pharmacology, School of Chinese Materia Medica, Beijing University of Chinese Medicine, Beijing, China, bucm.edu.cn; ^3^ Department of Oncology and Hematology, Dongzhimen Hospital, Beijing University of Chinese Medicine, Beijing, China, bucm.edu.cn; ^4^ School of Life Sciences, Beijing University of Chinese Medicine, Beijing, China, bucm.edu.cn; ^5^ Department of Chemistry, Tsinghua University, Beijing, China, tsinghua.edu.cn; ^6^ Beijing Research Institute of Chinese Medicine, Beijing University of Chinese Medicine, Beijing, China, bucm.edu.cn

**Keywords:** 4-methylesculetin, hepatic glucose metabolism, HepG2, insulin resistance, oxidative stress

## Abstract

**Background:**

Type 2 diabetes mellitus (T2DM) is a complex metabolic disorder characterized by elevated blood glucose.

**Objective:**

This study is aimed at evaluating the efficacy of 4‐methylesculetin in mitigating insulin resistance (IR) in HepG2 cells, thereby identifying the underlying mechanisms.

**Methods:**

An HepG2 cell insulin resistance (IR‐HepG2) model was established using high glucose and high insulin. Cell viability was evaluated via the CCK‐8 test to ascertain the safety dosage, whereas the impact of 4‐methylesculetin on glucose metabolism was investigated by quantifying glucose uptake and glycogen levels. The impact of 4‐methylesculetin on oxidative stress within the IR‐HepG2 model was assessed through the analysis of reactive oxygen species (ROS), malondialdehyde (MDA), superoxide dismutase (SOD), and glutathione peroxidase (GSH‐PX). To investigate the underlying mechanisms, Western blot analysis was performed to determine the protein expression levels of key molecules involved in insulin signaling and oxidative stress, including p‐AMPK, AMPK, SIRT1, NOX4, p‐AKT, AKT, p‐GSK3*β*, GSK3*β*, p‐FOXO1, FOXO1, p‐GYS1, GYS1, PEPCK, GLUT2, and G6Pase.

**Results:**

In the IR‐HepG2 model, 4‐methylesculetin treatment significantly enhanced glucose consumption and glycogen synthesis (*p* < 0.01). It also markedly alleviated oxidative stress by increasing the activities of antioxidant enzymes SOD and GSH‐PX (*p* < 0.05 or *p* < 0.01), and reducing the levels of ROS and MDA (*p* < 0.01). Western blot analysis revealed that these beneficial effects were mediated through the activation of the AMPK/FOXO1 and PI3K/AKT/GSK3*β* pathways, as well as the activation of SIRT1, which led to the suppression of NOX4.

**Conclusions:**

4‐Methylesculetin may ameliorate IR in HepG2 cells by improving glucose metabolism via AMPK/FOXO1 and PI3K/AKT/GSK3*β* pathways and attenuating oxidative stress via SIRT1/NOX4 axis. In addition, 4‐methylesculetin has the potential to be a therapeutic agent for T2DM.

## 1. Introduction

Type 2 diabetes mellitus (T2DM) is an increasing global health problem, and it is one of the most prevalent chronic noncommunicable diseases worldwide [1, 2] T2DM significantly affects patients, families, and society, imposing considerable burdens on individuals and communities [3]. Insulin resistance (IR) is a significant pathogenic mechanism in the development of and a primary contributor to its associated complications [4, 5]. Numerous factors, such as lifestyle choices, environmental conditions, and genetic susceptibility, might influence the onset of IR [6].

Oxidative stress is a critical factor in the development of IR [7]. It arises from an imbalance in the body brought on by either an excess of reactive oxygen species (ROS) produced by cells or a deficiency of antioxidants, which normally protect the body from excessive ROS [8]. Moreover, hyperglycemia and hyperlipidemia can promote significant ROS generation and chronic low‐grade inflammation [9]. This oxidative insult impairs insulin signaling pathways and damages insulin target organs, thereby exacerbating IR and the progression of T2DM. Phosphatidylinositol 3‐kinase (PI3K)/protein kinase B (AKT) pathway plays an important role in insulin signaling pathway, which is considered as the key regulator relevant to gluconeogenesis and glycogen synthesis. Impaired PI3K/AKT pathway in diabetes is one of the main mechanisms of IR induced by the increased level of ROS [10]. Furthermore, the observation that NADPH oxidase 4 (NOX4) inhibitors can enhance insulin sensitivity suggests that suppressing NOX4 holds potential in preventing IR [11].

As a key metabolic energy sensor, AMP‐activated protein kinase (AMPK) activation confers a therapeutic benefit against T2DM by lowering circulating glucose and lipid levels and improving insulin sensitivity [12]. Peroxisome proliferator‐activated receptor gamma (PPAR*γ*) is a key regulator of glucose metabolism and a well‐established target for enhancing insulin sensitivity [13]. It acts by upregulating multiple components within the glucose transporter 4 (GLUT4) signaling cascade, thereby stimulating glucose uptake [13]. Scopoletin enhances insulin signaling by increasing PPAR*γ* expression in adipocytes and stimulating AKT phosphorylation in insulin‐resistant HepG2 cells [14]. The forkhead box protein O1 (FOXO1) and peroxisome proliferator‐activated receptor gamma coactivator 1‐alpha (PGC‐1*α*) signaling axis is a key transcriptional pathway governing gluconeogenesis [15]. In brief, in response to fasting signals, FOXO1 is activated and translocates into the nucleus, where it associates with its cofactor PGC‐1*α* [16]. This complex then binds to the promoters of key gluconeogenic enzymes, such as phosphoenolpyruvate carboxykinase (PEPCK) and glucose‐6‐phosphatase (G6Pase), to drive their expression. Conversely, activated AMPK counteracts this process by facilitating the PI3K/AKT signaling pathway, which leads to the phosphorylation and inactivation of FOXO1, thereby suppressing its ability to transactivate gluconeogenic genes [15]. Furthermore, AMPK has been demonstrated to ameliorate oxidative stress and hepatic IR through the inhibition of NADPH oxidase‐mediated intracellular ROS production and the activation of sirtuin 1 (SIRT1) [17].

Coumarins are secondary metabolites that are widely distributed in natural plants. Research indicates that coumarin and its derivatives possess anti‐inflammatory and antioxidant properties that enhance insulin signaling and aid in the restoration of damaged pancreatic *β*‐cells [18, 19]. Moreover, specific coumarin derivatives have been shown to exert antidiabetic effects by targeting key metabolic regulators. For instance, osthole has been demonstrated to increase AMPK phosphorylation in C2C12 cells [12]. 4‐Methylesculetin (6,7‐dihydroxy‐4‐methylcoumarin, 4‐ME) was first isolated from *Artemisia annua* L. [20]. By improving the body′s capacity to metabolize glucose, lowering IR and reducing the accumulation of adipose tissue and liver lipid deposits, 4‐ME has been demonstrated to treat metabolic syndrome [21]. Additionally, 4‐ME has been demonstrated to lessen colon oxidative stress and large intestine colitis [22, 23]. In our preliminary work, 4‐methylesculetin was identified as a potential active component of Xiasangju against T2DM using surface plasmon resonance (SPR)‐LC‐MS technology [17]. Preliminary affinity assays indicated that 4‐methylesculetin exhibits high affinity for AMPK. However, its systematic effects on oxidative stress and glucose metabolism, along with the underlying mechanisms, remain to be fully elucidated. Therefore, this study established an IR‐HepG2 cell model and utilized 4‐ME as the test drug, aiming to evaluate its ameliorative effects on glucose metabolism and oxidative stress, and to deeply investigate the potential mechanisms at the molecular level.

## 2. Materials and Methods

### 2.1. Cell Culture and Treatment

**Figure 1:**
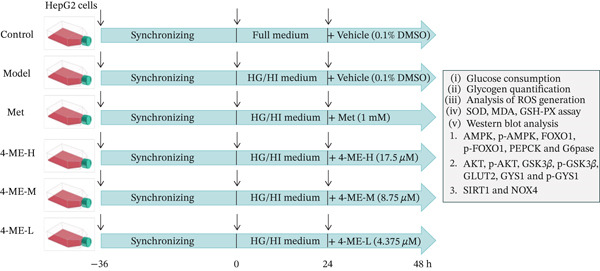
Experimental workflow for assessing 4‐ME in IR‐HepG2 cell model.

Human hepatocellular carcinoma cell line HepG2 cells were obtained from Procell Life Science & Technology Co. Ltd. (Wuhan, China; Cat. No. CL‐0103). The cells were cultured in accordance with the following parameters: 37°C in a 5% CO_2_ atmosphere in MEM (with NEAA) basal medium (Procell Life Science & Technology Co. Ltd, Wuhan, China; Cat. No. PM150410), 10% heat‐inactivated fetal bovine serum (Vazyme Biotech Co. Ltd, Nanjing, China; Cat. No. F101), 100 mg/mL penicillin and 100 mg/mL streptomycin (Thermo Fisher Scientific, United States; Cat. No. 15140122). The control group, model group, positive control group (Met, 1 mM) [24], high dose of 4‐ME group (4‐ME‐H, 17.5 *μ*M), medium dose of 4‐ME group (4‐ME‐M, 8.75 *μ*M), and low dose of 4‐ME group (4‐ME‐L, 4.375 *μ*M) were the six groups into which HepG2 cells were divided. An in vitro IR model of HepG2 cells was created, apart from the control group that received full media. The original medium was then taken out, and different medications were added for additional testing. HepG2 cells with a passage number range of 3–8 were employed in this study. The experimental design is shown in Figure 1.

### 2.2. Induction of Insulin‐Resistant HepG2 Cells

In this experiment, high concentrations of insulin and high glucose were used to establish an in vitro HepG2 IR cell model. The stability of the resulting construct was then evaluated [25]. Briefly, the HepG2 cells were subjected to a 12‐h starvation period in serum‐free medium. Then, different insulin (Novo Nordisk (China) Pharmaceuticals Co. Ltd, Tianjin, China; Batch No. 2023072402) concentrations (1 × 10^−4^, 1 × 10^−5^, 1 × 10^−6^, 1 × 10^−7^ and 1 × 10^−8^ mol/L) were added to a medium that contained 25 mmol/L glucose (Shanghai Yuanye Bio‐Technology Co. Ltd, Shanghai, China; Cat. No. S11022). At 24, 48, and 72 h, the effects on glucose consumption and HepG2 cell proliferative viability were evaluated accordingly. The IR‐HepG2 model was conditioned on insulin concentrations and durations that minimized glucose consumption without influencing cellular proliferation. Furthermore, an investigation was carried out to ascertain the stability of the model in HepG2 cells.

### 2.3. Cell Viability Assay

4‐Methylesculetin (98% purity) was purchased from Shanghai yuanye Bio‐Technology Co., Ltd (Shanghai, China; Cat. No. B24014). Cell counting kit‐8 (CCK‐8) assay (NCM Biotech, Suzhou, China; Cat. No. C6005) was used to estimate cell viability. HepG2 cells were cultivated for 24 h after being injected into 96‐well plates at a density of 1 × 10^4^ cells per well. They were then incubated for 24, 48, and 72 h with different doses of 4‐ME while 0.1% dimethyl sulfoxide (DMSO) (Sigma‐Aldrich, United States; Cat. No. 472301) was present. In the drug‐treated group, 100 *μ*L of 4‐ME solution at different concentrations was added to each well, and in the control group, 100 *μ*L of 0.1% DMSO was added to each well, and six parallel wells were set up in each group. After incubation, 10 *μ*L CCK‐8 solution was added to each well and incubated at 37°C for 0.5 h. Absorbance at 450 nm wavelength was determined using SpectraMax i3X multifunctional microplate reader (Molecular Devices, United States) and cell viability (%) was calculated.

### 2.4. Measurement of Glucose Consumption

HepG2 cells (2 × 10^6^ cells/well) were trypsinized with 0.25% trypsin (Thermo Fisher Scientific, United States; Cat. No. 25200056) to prepare a single‐cell suspension. The culture medium was then collected and centrifuged at 1000 rpm for 10 min. The resulting supernatant was collected for glucose measurement. According to the manufacturer′s instructions, glucose consumption was determined using a Glucose Assay Kit (Jiancheng Bioengineering Institute, Nanjing, China; Cat. No. A154‐1‐1).

### 2.5. Glycogen Quantification

HepG2 cells (2 × 10^6^ cells/well) were trypsinised with 0.25% trypsin and a single cell suspension was prepared, the cell precipitates were washed with PBS, an alkali solution was added, and the glycogen assay solution was prepared by boiling in a water bath for 20 min and cooling under running water. Following the manufacturer′s instructions, the amount of glycogen was measured using a glycogen content assay kit from Jiancheng Bioengineering Institute (Nanjing, China, Cat. No. A043‐1‐1).

### 2.6. Analysis of ROS Generation

The assessment of ROS generation was carried out using DCFH‐DA (Jiancheng Bioengineering Institute, Nanjing, China; Cat. No. E004‐1‐1). HepG2 cells were evenly seeded in 6‐well plates at a density of 2 × 10^6^ cells per well and cultured for 24 h. Posttreatment with high‐glucose, high‐insulin, and 4‐methylesculetin, the HepG2 cells were rinsed with PBS. Subsequently, they were incubated with 10 *μ*M DCFH‐DA for 1 h at 37°C in the dark. After that, the cells were collected, washed again with PBS, and resuspended in PBS. A BD FACS Calibur flow cytometer (Becton, Dickinson and Company, United States) was used to analyze HepG2 cells.

### 2.7. Measurement of Superoxide Dismutase (SOD) and Glutathione Peroxidase (GSH‐PX) Activities

The culture supernatant was removed, and the cells were trypsinized with 0.25% trypsin at room temperature for 1–2 min. The digestion was terminated by adding fresh culture medium, and the cells were gently resuspended. The cell suspension was centrifuged at 1000 rpm for 10 min, and the supernatant was discarded. The pellet was washed 1–2 times with PBS, with centrifugation at 1000 rpm for 10 min after each wash. After the final wash, the cell pellet was collected and lysed by adding an appropriate amount of lysis buffer (sufficient to cover the cells) for 30–40 min. The resulting lysate was used directly for subsequent assays. According to the manufacturer′s instructions, SOD activity was measured using an assay kit (Jiancheng Bioengineering Institute, Nanjing, China; Cat. No. A001‐3‐2), and GSH‐PX activity was determined using a corresponding kit (Jiancheng Bioengineering Institute, Nanjing, China; Cat. No. A005‐1‐2).

### 2.8. Measurement of Malondialdehyde (MDA) Level

The culture supernatant was removed, and the cells were trypsinized with 0.25% trypsin at room temperature. Subsequently, the cells were transferred to a centrifuge tube using a pipette. Following the manufacturer′s instructions, the level of MDA was determined using a kit from Jiancheng Bioengineering Institute (Nanjing, China; Cat. No. A003‐4‐1).

### 2.9. Western Blot Analysis

HepG2 cells were cultured at a concentration of 2 × 10^5^ cells/well into 6‐well culture plates. HepG2 cells were seeded in 6‐well plates at a density of 2 × 10^5^ cells/well. After adherence, the IR model was established, and the cells were subsequently grouped and treated according to the methods described in Sections 2.1 and 2.2. The cells were treated for 24 h, and the experiment was repeated three times. After being incubated for 24 h, the cells were taken out and lysed with RIPA buffer (NCM Biotech, Suzhou, China; Cat. No. WB3100), which contains both phosphatase and protease inhibitors (NCM Biotech, Suzhou, China; Cat. No. P003). A bicinchoninic acid (BCA) protein assay kit (NCM Biotech, Suzhou, China; Cat. No. WB6501) was used to quantify the total protein concentration of each sample after the supernatant was collected. The samples were dissolved in 5 × protein sample loading buffer (NCM Biotech, Suzhou, China; Cat. No. WB2001), vortexed, and boiled at 100°C for 10 min. Equal amounts of protein (10 *μ*g) were separated by SDS‐PAGE using an 8–10% gel (Shanghai Yeasen Biotechnology Co., Ltd, Shanghai, China), first run at 110 V for 10 min through the stacking gel, and then at 200 V for 25–30 min through the separating gel. The separated proteins were subsequently transferred to a PVDF membrane (0.22 *μ*m; Merck Millipore, Germany; Cat. No. ISEQ00010) at a constant current of 400 mA for 40–50 min. A protein‐free quick sealing solution (Servicebio, Wuhan, China; Cat. No. G2052) was then used to block the membrane for 30 min at room temperature. The PVDF membrane was incubated with primary antibodies, including anti‐SIRT1 (1:1000, 13161‐1‐AP, Proteintech), antiglycogen synthase 1 (GYS1) (1:1000, 10566‐1‐AP, Proteintech), antiphosphorylated glycogen synthase 1 (p‐GYS1) (1:1000, ab81230, Abcam), antiphosphorylated forkhead box O1 (p‐FOXO1) (1:1000, ab259337, Abcam), anti‐FOXO1 (1:1000, ab179450, Abcam), anti‐PEPCK (1:1000, 16754‐1‐AP, Proteintech), anti‐NOX4 (1:1000, 67681‐1‐Ig, Proteintech), anti‐GLUT2 (1:1000, 20436‐1‐AP, Proteintech), anti‐AMPK (1:1000, ab207442, Abcam), antiphosphorylated‐AMP‐activated protein kinase (p‐AMPK) (1:2000, ab23875, Abcam), antiphosphorylated protein kinase B (p‐AKT) (1:1000, ab192623, Abcam), anti‐AKT (1:1000, ab179463, Abcam), antiphosphorylated glycogen synthase kinase‐3*β* (p‐GSK3*β*) (1:1000, 9323, CST), antiglycogen synthase kinase‐3*β* (GSK3*β*) (1:1000, ab280376, Abcam), anti‐G‐6pase (1:1000, ab31905, Abcam), and anti‐*β*‐actin (1:10000, 66009‐1‐Ig, Proteintech) at 4°C for 12 h. The PVDF membrane was subjected to a three‐stage washing process with TBST buffer (Servicebio, Wuhan, China; Cat. No. G0001), each stage lasting 10 min. The PVDF membrane was then treated for 1 h at room temperature with HRP‐conjugated AffiniPure goat antirabbit or antimouse IgG (H + L) (1:5000, Proteintech). The PVDF membrane was then washed with TBST buffer on three further occasions, each stage lasting 10 min. Protein strips on PVDF membranes were scanned with chemiluminescent reagent (NCM Biotech, Suzhou, China; Cat. No. P10100) and detected by the ChemiDoc MP imaging system (Bio‐Rad, United States). Visualization was conducted with Image J software v1.8.0 (National Institutes of Health, Rockville, Maryland, United States). Protein bars were normalized with *β*‐actin, and all results were independently repeated three times.

### 2.10. Statistical Analysis

The statistical analysis of all data in this study was performed using SPSS 22.0 and GraphPad Prism 8 software. The resulting data are presented as means ± standard deviations (SDs). One‐way analysis of variance (ANOVA) was applied when the data from all groups followed a normal distribution and had homogeneity of variances; the least significant difference (LSD) test was then used for post hoc pairwise comparisons. If the data did not follow a normal distribution or the variances were unequal, nonparametric tests were employed. *p* < 0.05 was considered to indicate statistical significance.

Human HepG2 cells were synchronized, serum‐starved, and treated as indicated. The Control group was cultured in full medium. IR was induced in the other groups using high‐glucose/high‐insulin (HG/HI) medium. The Model group received vehicle, whereas treatment groups received metformin (1 mM) or 4‐ME at the indicated concentrations. After 24 h, cells were harvested for analysis.

## 3. Results

### 3.1. Establishment of Insulin‐Resistant HepG2 Cell Model

Cell viability was determined by the CCK‐8 assay. As shown in Figures 2a, 2b, and 2c, HepG2 cells treated with 10^−4^ mol/L insulin for 24, 48, and 72 h showed a significant reduction in cell viability (*p* < 0.05 or *p* < 0.01). A similar trend was observed with 10^−5^ mol/L insulin, although the decrease was only statistically significant after a 72‐h treatment period.

Figure 2Establishment of the IR‐HepG2 cell model. Viability of HepG2 cells treated with the indicated concentrations of insulin for (a) 24 h, (b) 48 h and (c) 72 h, as assessed by CCK‐8 assay. Glucose consumption in HepG2 cells treated with the indicated concentrations of insulin for (d) 24 h, (e) 48 h, and (f) 72 h. (g) Stabilizing effects of the IR‐HepG2 cell model. Data were shown as mean ± SD.  ^∗^ *p* < 0.05,  ^∗∗^ *p* < 0.01 versus zero M insulin **(** *n* = 3, from three independent experiments). Statistical significance was determined by SPSS for comparisons against the 0 *μ*M 4‐Methylesculetin group. ∗*p* < 0.05, ∗∗*p* < 0.01.

**Figure:**
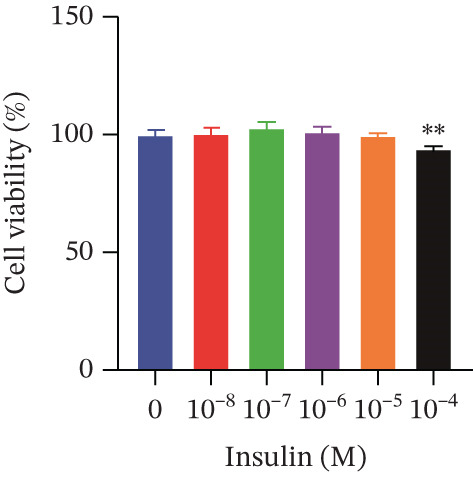
(a)

**Figure:**
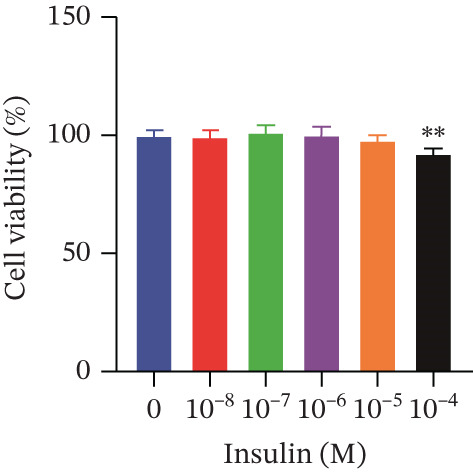
(b)

**Figure:**
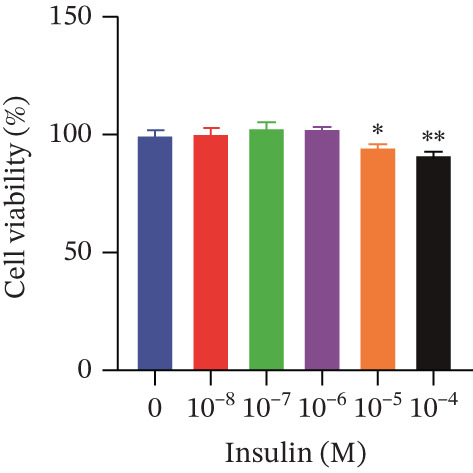
(c)

**Figure:**
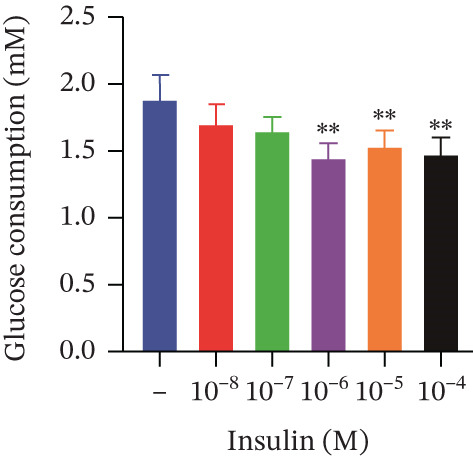
(d)

**Figure:**
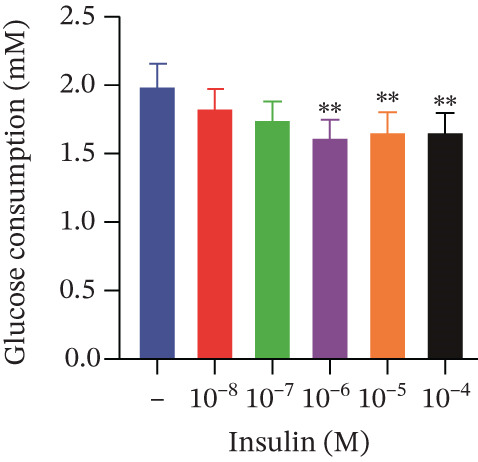
(e)

**Figure:**
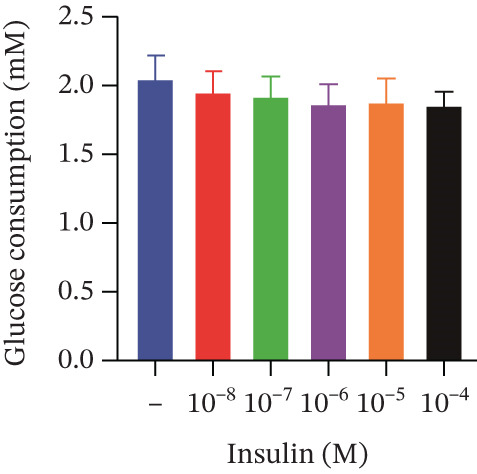
(f)

**Figure:**
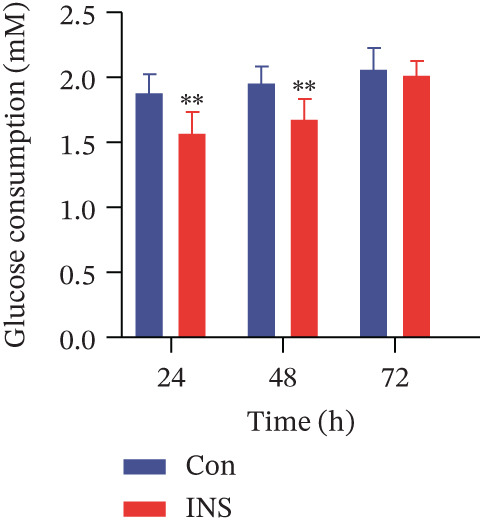
(g)

As shown in Figures2d, 2e, and 2f, when HepG2 cells were given 10^−6^, 10^−5^, and 10^−4^ mol/L of insulin for 24 and 48 h, their glucose consumption decreased significantly (*p* < 0.01) compared with the control group. However, no statistically significant difference was observed in glucose consumption when the cells were treated with insulin for 72 h. The glucose consumption at 10^−8^ and 10^−7^ mol/L insulin, administered to the cells for 24, 48, and 72 h, showed no statistically significant difference. The optimal insulin concentration for establishing the IR‐HepG2 model in this experimental procedure was determined to be 10^−6^ mol/L, with a duration of action of 24 h.

Following a 24‐h incubation of 10^−6^ mol/L insulin to HepG2 cells, glucose intake was measured at predefined intervals to assess the durability of the IR‐HepG2 cell model. Figure 2g shows that incubation with 10^−6^ mol/L insulin significantly suppressed glucose consumption in HepG2 cells compared with the control at the 24‐ and 48‐h time points (*p* < 0.01). By 72 h, however, glucose consumption in the insulin‐exposed cells was no longer significantly different from that of the control. These findings indicated that the IR‐HepG2 cell model established by this method has an effective duration of 48 h.

### 3.2. Determination of the Administrating Dose of 4‐Methylesculetin

To determine the administrating dose of 4‐ME, the safe dosage should be identified firstly. We assessed the viability of HepG2 cells using the CCK‐8 assay. As shown in Figure 3, cell viability was significantly reduced in the 35 *μ*M, 70 *μ*M and 140 *μ*M 4‐ME groups at 48 h, as well as in the 160 *μ*M group at 24, 48, and 72 h, compared with the control group (*p* < 0.05 or *p* < 0.01). Results indicated that the viability of HepG2 cells decreased progressively with increasing concentrations of 4‐ME and longer incubation times. Based on these findings, we selected 17.5 *μ*M, 8.75 *μ*M, and 4.375 *μ*M as the high (4‐ME‐H), medium (4‐ME‐M), and low (4‐ME‐L) doses of 4‐ME, respectively, for subsequent experiments.

Figure 3Effect of 4‐methylesculetin on IR‐HepG2 cell viability. (a) 24 h, (b) 48 h, (c) 72 h cell viability in the CCK‐8 assay. Data were shown as mean ± SD.  ^∗^ *p* < 0.05,  ^∗∗^ *p* < 0.01 versus. 0 *μ*M 4‐Methylesculetin (*n* = 3, from three independent experiments). Statistical significance was determined by SPSS for comparisons against the 0 M 4‐Methylesculetin group. ∗*p* < 0.05, ∗∗*p* < 0.01.

**Figure:**
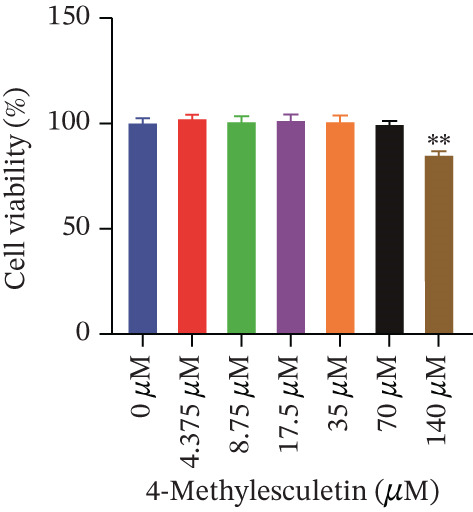
(a)

**Figure:**
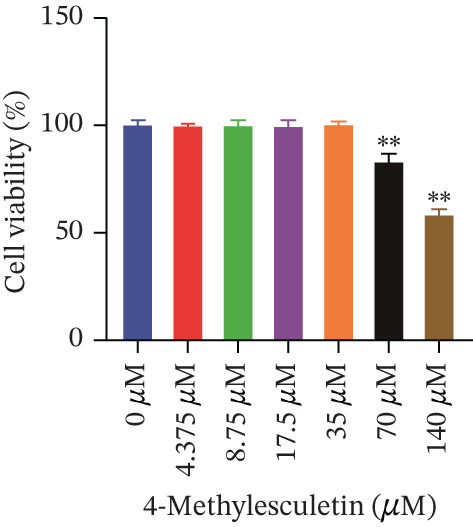
(b)

**Figure:**
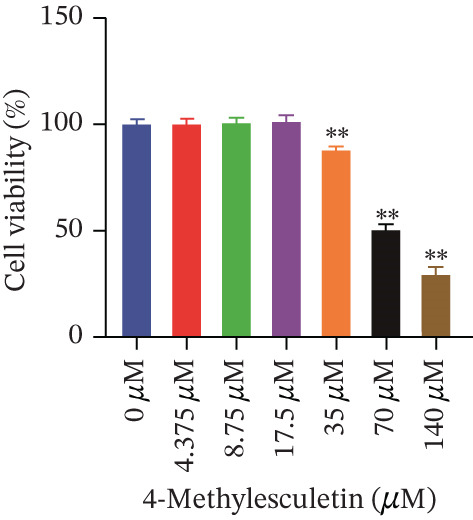
(c)

### 3.3. 4‐ME Promoted Activation of AMPK and Prevented Gluconeogenesis in IR‐HepG2 Cells

Hepatic gluconeogenesis is frequently elevated in patients with T2DM [26]. In the present study, glucose consumption was significantly lower in the model group than in the control group (*p* < 0.01). Treatment with 4‐ME (4‐ME‐H, 4‐ME‐M, and 4‐ME‐L groups) and metformin (met group) significantly restored glucose consumption compared with the Model group (*p* < 0.01) (Figure 4a), suggesting an improvement in IR.

Figure 4Effect of 4‐ME on gluconeogenesis. (a) Glucose consumption; (b) representative images of Western blot; (c) the level of FOXO1 phosphorylation; (d) the level of PEPCK protein expression; (e) the level of AMPK phosphorylation; (f) the level of G6Pase protein expression. Data were shown as mean ± SD.  ^∗^ *p* < 0.05,  ^∗∗^ *p* < 0.01 versus control group; ^#^ *p* < 0.05, ^##^ *p* < 0.01 versus model group, *n* = 3 independent experiments for each group.

**Figure:**
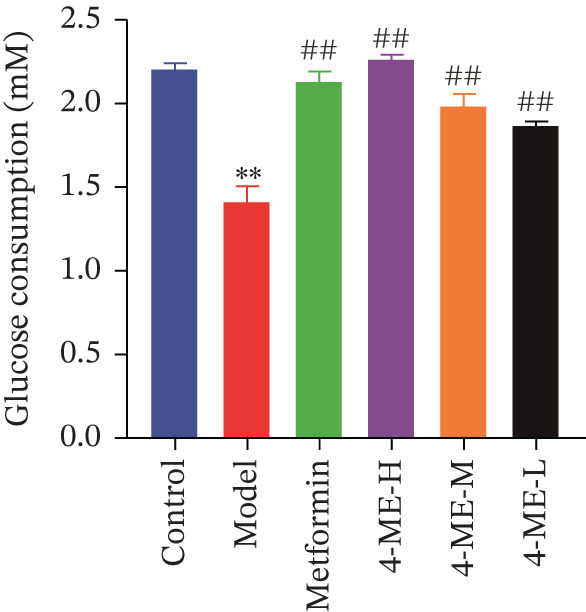
(a)

**Figure:**
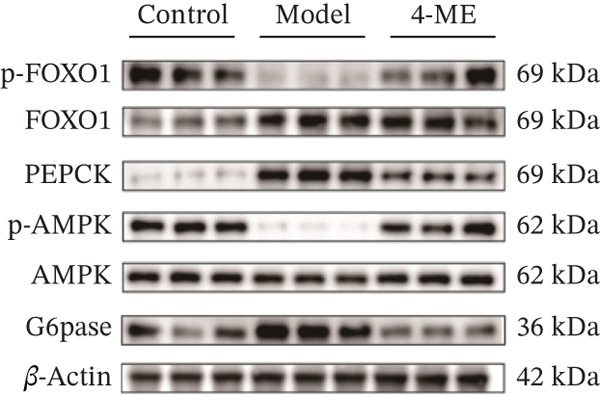
(b)

**Figure:**
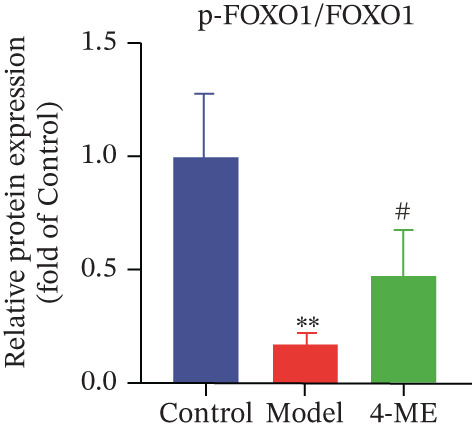
(c)

**Figure:**
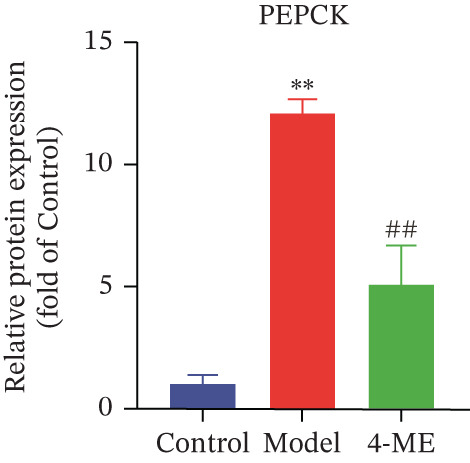
(d)

**Figure:**
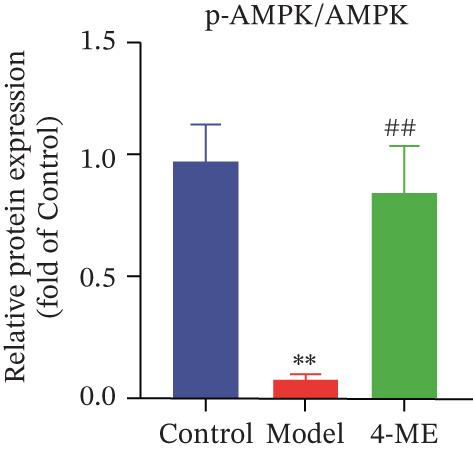
(e)

**Figure:**
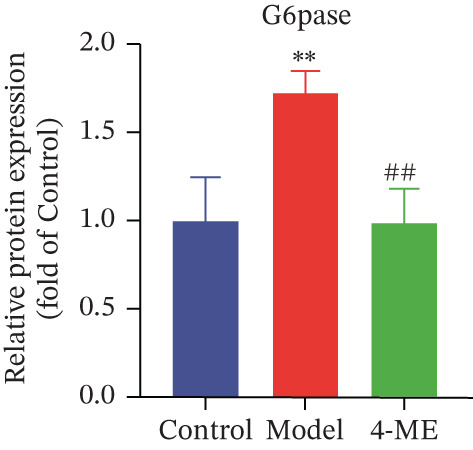
(f)

To investigate the underlying mechanism, we examined the AMPK/FOXO1 pathway. As shown in Figures 4b, 4c, 4d, 4e, and 4f, the Model group exhibited a marked decrease in the protein expression ratios of p‐AMPK/AMPK and p‐FOXO1/FOXO1 (*p* < 0.01). Conversely, 4‐ME treatment significantly upregulated these phosphorylation levels (*p* < 0.01). Consistent with the activation of this pathway, which is known to suppress gluconeogenesis, the expression of the key gluconeogenic enzymes PEPCK and G6Pase was significantly elevated in the model group but was markedly suppressed by 4‐ME (*p* < 0.01). Taken together, these results indicate that 4‐ME inhibits hepatic gluconeogenesis in HepG2 cells by activating the AMPK/FOXO1 signaling pathway.

### 3.4. 4‐ME Promotes Glycogen Content and Synthesis in HepG2 Cells via Activating the PI3K/AKT Pathway

Recent studies have found a substantial correlation between IR and hepatic glycogen production, with T2DM animals exhibiting decreased hepatic glycogen synthesis and content [27, 28]. Consistent with these findings, our study revealed a significantly lower glycogen content in the model group compared with the control group (*p* < 0.01, Figure 5a). This defect was markedly rescued by treatment with 4‐ME (4‐ME‐H, 4‐ME‐M, and 4‐ME‐L groups) and metformin (met group), which significantly increased glycogen levels relative to the model group (*p* < 0.01).

Figure 5Effect of 4‐ME on glycogen synthesis. (a) hepatic glycogen. (b) Representative images of Western blot. (c) The level of GYS1 phosphorylation. (d) The level of GLUT2 protein expression. (e) The level of AKT phosphorylation. (f) The level of GSK3*β* phosphorylation. Data were shown as mean ± SD.  ^∗^ *p* < 0.05,  ^∗∗^ *p* < 0.01 versus control group; ^#^ *p* < 0.05, ^##^ *p* < 0.01 versus model group, *n* = 3 independent experiments for each group.

**Figure:**
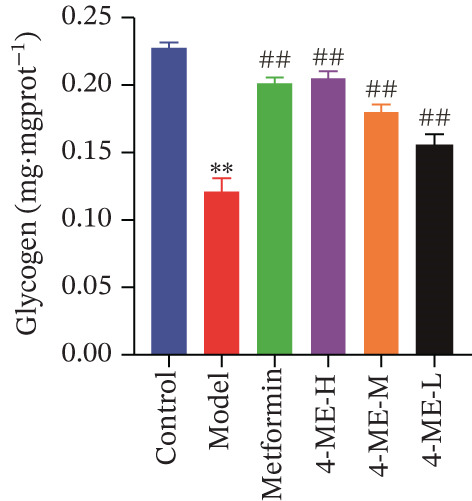
(a)

**Figure:**
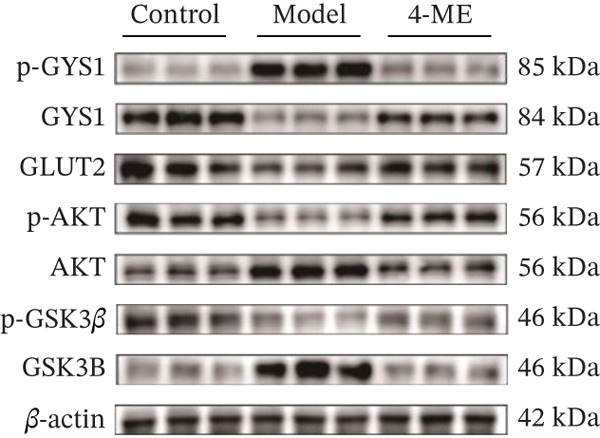
(b)

**Figure:**
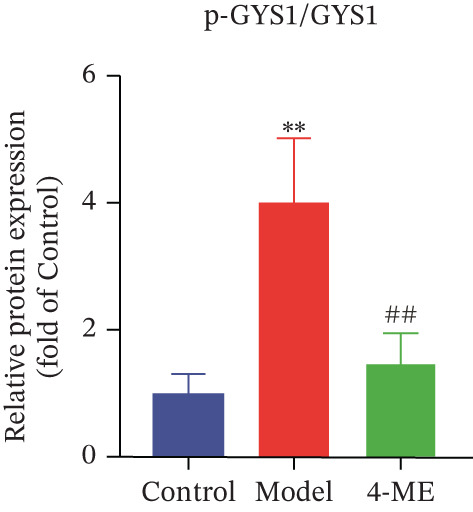
(c)

**Figure:**
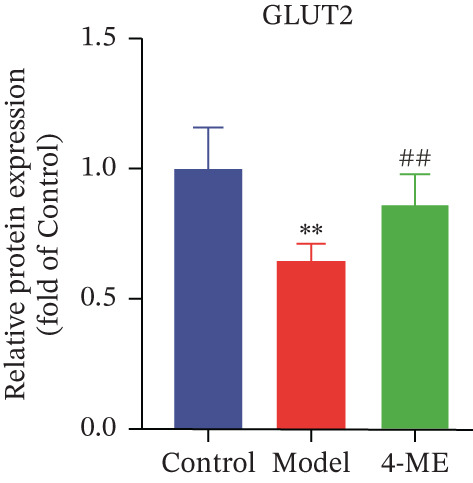
(d)

**Figure:**
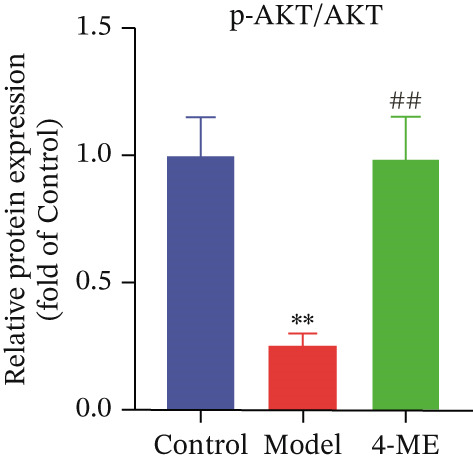
(e)

**Figure:**
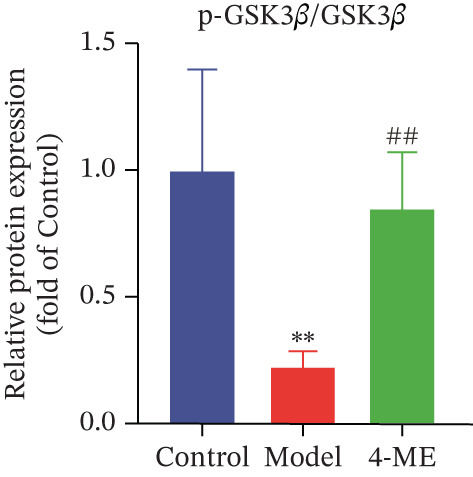
(f)

The PI3K/AKT pathway is the canonical insulin signaling pathway, and hepatic IR is usually associated with impaired insulin signaling [29]. One of the key enzymes in the glycogen manufacturing process is glycogen synthase (GS). Activated AKT phosphorylates and inhibits GSK3*β*, which decreases phosphorylation of GS and increases its activity, hence boosting glycogen production [30]. Furthermore, GLUT2 is a glucose transporter protein located on cell membranes. The liver absorbs glucose during elevated blood glucose levels and releases it as needed to maintain stable hepatic glucose metabolism [31].

Our results demonstrated that the model group exhibited a significant downregulation of the PI3K/AKT pathway, manifested as reduced phosphorylation of AKT and GSK3*β* (*p* < 0.01), along with a significant decrease in GLUT2 protein levels (*p* < 0.01). Concordantly, phosphorylation of GYS1 was significantly increased (*p* < 0.01), indicating its inactivation. Treatment with 4‐ME effectively mitigated these abnormalities, significantly enhancing the phosphorylation of AKT and GSK3*β*, upregulating GLUT2 expression, and reducing GYS1 phosphorylation (*p* < 0.01, Figures 5b, 5c, 5d, 5e, and 5f). In conclusion, these results demonstrate that 4‐ME promotes hepatic glycogen synthesis in IR‐HepG2 cells by activating the PI3K/AKT/GSK3*β* pathway and ameliorating the disruption of insulin signaling.

### 3.5. 4‐ME Inhibited Oxidative Stress and the Overexpression of the NOX4 Protein in IR‐HepG2 Cells

Oxidative stress and IR in the liver have been linked in recent research [32, 33], with elevated ROS recognized as a primary contributor to IR [34, 35]. In this study, ROS and MDA levels exhibited a notable elevation in the model group relative to the control group (*p* < 0.01). Concurrently, SOD and GSH‐PX levels demonstrated a discernible decline (*p* < 0.01). In comparison to the model group, the 4‐ME‐H, 4‐ME‐M, 4‐ME‐L, and met groups exhibited markedly reduced ROS and MDA levels (*p* < 0.01) and notably elevated SOD and GSH‐PX levels (*p* < 0.05 or *p* < 0.01) (Figures 6a, 6b, 6c, and 6d).

Figure 64‐Methylesculetin ameliorates oxidative stress in IR‐HepG2 cells. (a) ROS; (b) MDA; (c) SOD; (d) GSH‐PX; (e) representative images of Western blot; (f) the level of SIRT1 protein expression; (g) the level of NOX4 protein expression. Data were shown as mean ± SD.  ^∗^ *p* < 0.05,  ^∗∗^ *p* < 0.01 versus control group; ^#^ *p* < 0.05, ^##^ *p* < 0.01 versus model group, *n* = 3 independent experiments for each group.

**Figure:**
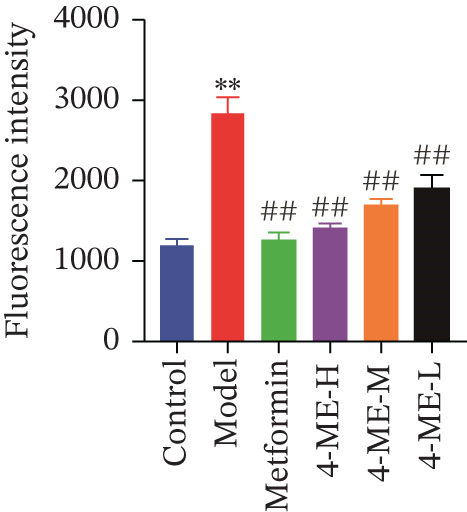
(a)

**Figure:**
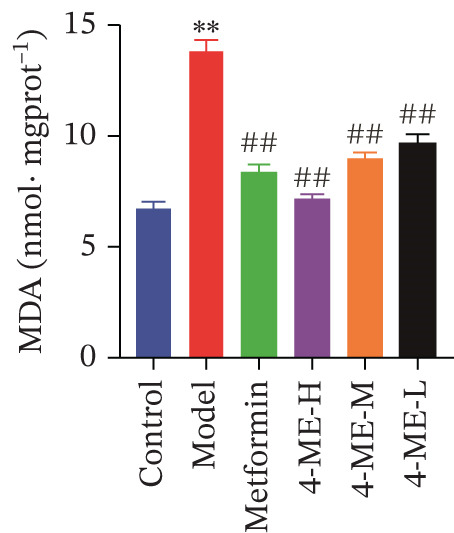
(b)

**Figure:**
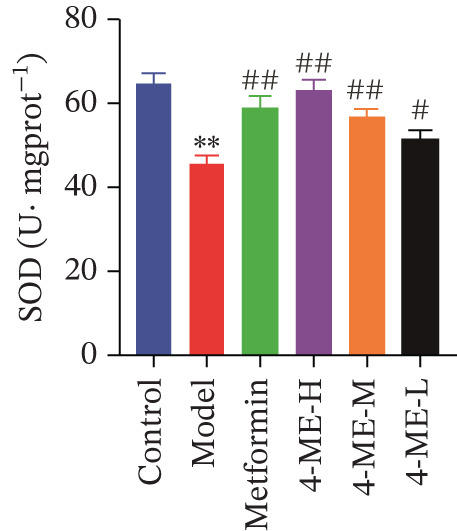
(c)

**Figure:**
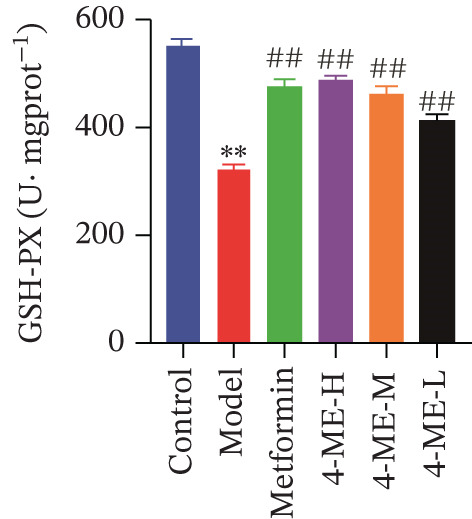
(d)

**Figure:**
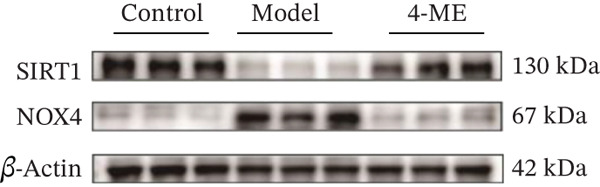
(e)

**Figure:**
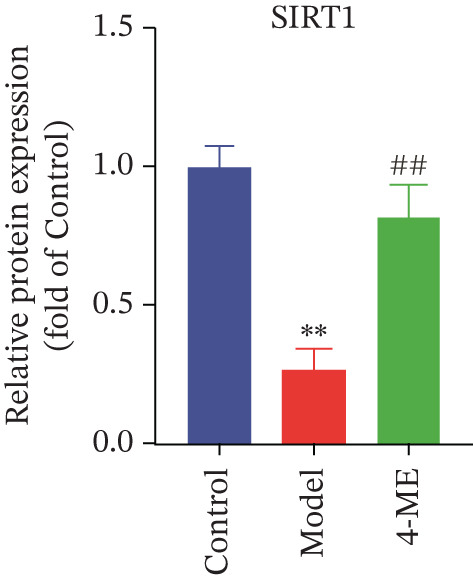
(f)

**Figure:**
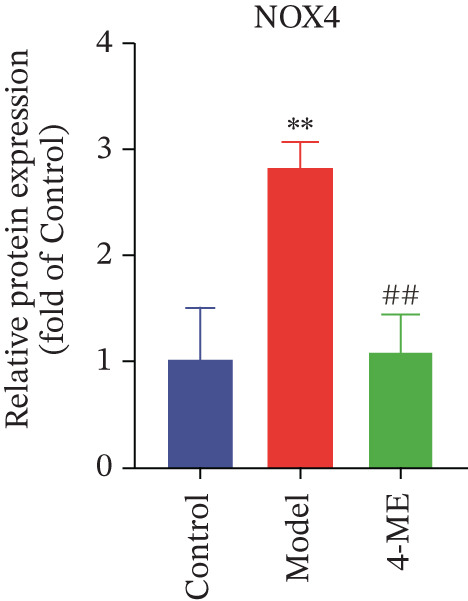
(g)

We further investigated the mechanistic basis for this antioxidant effect. One of the main sources of ROS generation is thought to be NADPH oxidases [36]. Hepatic IR is substantially linked to NOX4 [10]. In this study, 4‐ME treatment significantly upregulated the protein expression of SIRT1 while concurrently downregulating NOX4 expression (*p* < 0.01) (Figures 6e, 6f, and 6g). These findings indicate that 4‐ME alleviates oxidative stress and enhances hepatic insulin sensitivity, at least in part, by modulating the SIRT1/NOX4 pathway.

## 4. Discussion

The 10th edition of the International Diabetes Federation (IDF) Diabetes Atlas indicates that in 2024, 588.7 million adults (aged 20–79) globally have diabetes. By 2050, this number is projected to rise to 852.5 million, representing a 45% increase over 2024 [37]. Furthermore, 634.8 million adults (12.0%) globally have impaired glucose tolerance (IGT) in 2024, which puts them at higher risk of T2DM; this figure is projected to reach 846.5 million (12.9%) by 2050 [37]. This presents a significant public health concern and an onerous economic burden to the broader society. One early and important component in the etiology of T2DM is IR [38]. Abnormal hepatic glucose metabolism is one of the hallmarks of IR [39].

Our preliminary research has identified 4‐ME as a potential active component of Xiasangju in the treatment of T2DM [17]. 4‐ME has been shown to have important anti‐inflammatory and antioxidant qualities, and it has been used therapeutically to treat a number of illnesses, including metabolic syndrome [17]. The primary findings of this study are as follows (1) 4‐ME has the potential to reduce IR‐HepG2 gluconeogenesis, increase glycogen synthesis, and enhance hepatic glucose metabolism; (2) 4‐ME activates the AMPK and PI3K/AKT pathways of IR‐HepG2 cells, thereby improving IR; (3) 4‐ME alleviates the oxidative stress mediated by NOX4 in IR‐HepG2 cells, thereby ameliorating IR.

Stable blood glucose levels are controlled by the liver [40]. This is accomplished through glycogen synthesis and decomposition, which are essential mechanisms in blood glucose management [41]. Insulin regulates glucose metabolism in the liver by inhibiting gluconeogenesis and glycogen catabolism, which reduces glucose synthesis and the conversion of nonglucose substrates to glucose [42]. Additionally, it enhances the storage of glucose and promotes the synthesis of glycogen [43]. The main reasons of fasting hyperglycemia in T2DM patients are increased endogenous gluconeogenesis, decreased hepatic glycogen reserve, and improved gluconeogenesis [44, 45]. The enhancement of hepatic glycogen synthesis and the decrease in glucose production are considered essential therapeutic objectives in the treatment of diabetes mellitus [46].

PEPCK and G6Pase are two key rate‐limiting enzymes in hepatic gluconeogenesis [16]. In its active state, FOXO1 can upregulate the transcription of PEPCK and G6Pase; however, upon phosphorylation, it is exported from the nucleus, consequently losing its transcriptional activity and suppressing gluconeogenesis [47]. AMPK, acting as a cellular energy sensor, is an upstream regulator of FOXO1. By phosphorylating FOXO1, AMPK inhibits FOXO1‐mediated expression of gluconeogenic genes [48]. The results of this study demonstrate that 4‐methylesculetin activates AMPK, which in turn promotes the phosphorylation of FOXO1, ultimately inhibiting the protein expression of PEPCK and G6Pase. Through this mechanism, 4‐methylesculetin effectively regulates hepatic glucose metabolism and reduces the gluconeogenic level in IR‐HepG2 cells.

Hepatic IR has been demonstrated to result from disturbance of the insulin signaling system [49]. Insulin binds to its receptor to initiate the insulin signaling pathway, which phosphorylates IRS‐1 and activates the PI3K/AKT pathway [50]. The PI3K/Akt signaling pathway is essential for normal metabolic function in insulin target tissues, and its impairment is a fundamental cause of IR and the development of T2DM [51]. A key consequence of this impairment in T2DM is the overactivity of GSK3*β*, which contributes to increased glucose production and decreased insulin sensitivity [52]. Within the insulin signaling cascade, GSK3*β* is a critical enzyme regulated by Akt. Activated Akt phosphorylates GSK3*β*, which inhibits its kinase activity [51]. Under normal conditions, GSK3*β* phosphorylates and inhibits GS, thereby reducing hepatic glycogen synthesis and elevating blood glucose [53]. Furthermore, AKT stimulates glucose uptake into hepatocytes via the GLUT2 transporter [54]. Glycogen, the primary energy reserve in mammals, is synthesized by enzymes like GYS1, whose activity is negatively regulated by GSK3*β* phosphorylation across species [55, 56]. Our results demonstrate that in the IR‐HepG2 model, 4‐ME ameliorates hepatic IR by activating the PI3K/AKT signaling pathway. This leads to the phosphorylation and inhibition of GSK3*β*, which in turn reduces the phosphorylation of GS, ultimately promoting glycogen synthesis and restoring hepatic glucose metabolism.

A major contributing factor to hepatic IR in diabetics is oxidative stress [57]. HepG2 cells and mice with T2DM caused by a high‐fat diet and streptozotocin showed increased liver oxidative stress when treated with high‐glucose, according to a previous study [7, 58]. GSH, MDA, and SOD are the main biomarkers of oxidative stress. SOD serves as a crucial antioxidant enzyme, whereas GSH is vital for catalyzing hydrogen peroxide breakdown, making both of them central elements in the cellular defense system [59]. The increase in MDA concentration and the decrease in SOD activity indicate a state of oxidative stress [60]. This imbalance can damage pancreatic beta cell function and contribute to peripheral IR, thereby promoting the development of diabetes [60]. This study demonstrated that 4‐ME increased GSH and SOD levels while decreasing the MDA level in IR‐HepG2 cells, suggesting that 4‐ME may reduce oxidative stress in these cells. Multiple mechanisms contribute to oxidative stress in diabetic patients, with NADPH oxidases serving as the main sources of ROS [29]. Oxidative stress is caused in the diabetic liver by the activation of NOX4, a type of NADPH oxidase [61]. SIRT1 plays a pivotal role in the regulation of metabolic diseases, functioning as a sensor and regulator in response to oxidative stress. It lowers oxidative stress by triggering a number of downstream effector antioxidant signals, including endothelial NO synthase and NADPH oxidase control [62]. According to the results of this investigation, NOX4 expression rose whereas SIRT1 expression decreased in IR‐HepG2 cells. Alternatively, 4‐ME was observed to decrease NOX4 overexpression and boost SIRT1 expression. Conversely, 4‐ME was observed to elevate SIRT1 expression and mitigate NOX4 overexpression. 4‐ME mitigated oxidative stress in IR‐HepG2 cells by enhancing SIRT1 expression and reducing NOX4 overexpression in HepG2 cells.

This study demonstrates that 4‐ME effectively ameliorates hepatic IR in IR‐HepG2 cells through a multifaceted mechanism. Specifically, 4‐ME activates the AMPK/FOXO1 signaling axis, leading to the suppression of key gluconeogenic enzymes (PEPCK and G6Pase) and a consequent reduction in hepatic glucose output. Concurrently, it enhances the PI3K/AKT/GSK3*β* pathway, which promotes glycogen synthesis by alleviating the inhibitory phosphorylation of GS. Furthermore, 4‐ME mitigates oxidative stress, a critical contributor to IR, by upregulating SIRT1 expression and suppressing the ROS‐generating enzyme NOX4, thereby rebalancing cellular redox status. These coordinated actions on glucose metabolism and oxidative stress underscore the potential of 4‐ME as a promising therapeutic candidate for T2DM. However, several limitations must be addressed to translate these findings. The reliance on a single cell line model and the lack of in vivo confirmation limit the physiological relevance of our conclusions. Furthermore, a more precise dose‐response relationship across these pathways needs to be warranted. Consequently, future studies can focus on validating the efficacy of 4‐ME in animal models and delineating its comprehensive dose‐effect relationship, which are essential prerequisites for any future clinical application.

## 5. Conclusion

In conclusion, 4‐methylesculetin ameliorates hepatic IR by coordinately regulating glucose metabolism and alleviating oxidative stress through promoting glucose disposal via the PI3K/AKT/GSK3*β* pathway and suppressing gluconeogenesis via the AMPK/FOXO1 axis while concurrently mitigating oxidative damage through the SIRT1/NOX4 pathway. These integrated actions delineate a multifaceted cellular mechanism, establishing a solid theoretical foundation for future investigations.

**Nomenclature**
AKT: protein kinase B
AMPK: AMP‐activated protein kinase
ANOVA: one‐way analysis of variance
BCA: bicinchoninic acid
CCK‐8: cell counting kit‐8
DMSO: dimethyl sulfoxide
FOXO1: forkhead box O1
GLUT4: glucose transporter type 4
GSH‐PX: glutathione peroxidase
GSK3*β*
: glycogen synthase kinase‐3*β*
GS: glycogen synthase
GYS1: glycogen synthase 1
G6Pase: glucose‐6‐phosphatase
IDF: International Diabetes Federation
IGT: impaired glucose tolerance
HI: high‐insulin
HG: high‐glucose
IR: insulin resistance
LSD: least significant difference
MDA: malondialdehyde
4‐ME: 4‐methylesculetin
NOX4: NADPH oxidase 4
Nrf2: novel NF‐E2‐related factor 2
p‐AMPK: phosphorylated‐AMP‐activated protein kinase
p‐AKT: phosphorylated protein kinase B
PBS: phosphate buffered saline
PEPCK: phosphoenolpyruvate carboxykinase
p‐FOXO1: phosphorylated forkhead box O1
p‐GSK3*β*
: phosphorylated glycogen synthase kinase‐3*β*
p‐GYS1: phosphorylated glycogen synthase 1
PGC‐1*α*
: peroxisome proliferator‐activated receptor gamma coactivator 1‐alpha
PI3K: phosphatidylinositol 3‐kinase
PPAR*γ*
: peroxisome proliferator‐activated receptor *γ*
SDS‐PAGE: sodium dodecyl sulfate‐polyacrylamide gel electrophoresis
ROS: reactive oxygen species
SDs: standard deviations
SIRT1: sirtuin 1
SOD: superoxide dismutase
SPR: surface plasmon resonance
T2DM: Type 2 diabetes mellitus

## Author Contributions

**Xiaohua Su:** methodology, formal analysis, funding acquisition. **Yuhang Du:** writing, original draft, methodology, investigation, and conceptualization. **Yang Yang:** visualization, resources, methodology. **Yige Zhao:** visualization, resources. **Hongbin Zhao:** visualization, resources. **Jiamei Xie:** investigation and methodology. **Ziyi Shan:** software and visualization. **Menglu Wang:** methodology and software. **Zhiyun Huang:** visualization and resources. **Wanxin Fu:** data curation. **Anfeng Wan:** supervision. **Yongcheng An:** supervision, project administration, methodology. **Baosheng Zhao:** writing—review and editing, supervision, project administration, funding acquisition, conceptualization.

## Funding

This study was supported by Baosheng Zhao (82274166) and Xiaohua Su (2022‐XQ‐YFB‐008).

## Ethics Statement

HepG2 cells were obtained from Procell Life Science & Technology Co., Ltd. (Wuhan, China). All experimental conditions were approved by the Beijing University of Chinese Medicine.

## Consent

All authors agree to publish this article.

## Conflicts of Interest

The authors declare no conflicts of interest.

## Data Availability

The data will be made available upon request.
